# Post-anthesis Relationships Between Nitrogen Isotope Discrimination and Yield of Spring Wheat Under Different Nitrogen Levels

**DOI:** 10.3389/fpls.2022.859655

**Published:** 2022-03-17

**Authors:** Zechariah Effah, Lingling Li, Junhong Xie, Benjamin Karikari, Jinbin Wang, Min Zeng, Linlin Wang, Solomon Boamah, Jagadabhi Padma Shanthi

**Affiliations:** ^1^State Key Laboratory of Arid Land Crop Science, Lanzhou, China; ^2^College of Agronomy, Gansu Agricultural University, Lanzhou, China; ^3^Council for Scientific and Industrial Research (CSIR)-Plant Genetic Resources Research Institute, Bunso, Ghana; ^4^Department of Crop Science, Faculty of Agriculture, Food and Consumer Sciences, University for Development Studies, Tamale, Ghana; ^5^Biocontrol Engineering Laboratory of Crop Diseases and Pests of Gansu Province, College of Plant Protection, Gansu Agricultural University, Lanzhou, China; ^6^Action for Climate and Environment Program, Dr. Reddy’s Foundation, Hyderabad, India

**Keywords:** N remobilization, harvest index, ^15^N labeling, nitrogen uptake, sustainable production

## Abstract

Wheat grain yield and nitrogen (N) content are influenced by the amount of N remobilized to the grain, together with pre-anthesis and post-anthesis N uptake. Isotopic techniques in farmed areas may provide insight into the mechanism underlying the N cycle. ^15^N-labeled urea was applied to microplots within five different fertilized treatments 0 kg ha^–1^ (N1), 52.5 kg ha^–1^ (N2), 105 kg ha^–1^ (N3), 157.5 kg ha^–1^ (N4), and 210 kg ha^–1^ (N5) of a long-term field trial (2003–2021) in a rainfed wheat field in the semi-arid loess Plateau, China, to determine post-anthesis N uptake and remobilization into the grain, as well as the variability of ^15^N enrichment in aboveground parts. Total N uptake was between 7.88 and 29.27 kg ha^–1^ for straw and 41.85 and 95.27 kg ha^–1^ for grain. In comparison to N1, N fertilization increased straw and grain N uptake by 73.1 and 56.1%, respectively. Nitrogen use efficiency (NUE) and harvest index were altered by N application rates. The average NUE at maturity was 19.9% in 2020 and 20.01% in 2021; however, it was usually higher under the control and low N conditions. The amount of ^15^N excess increased as the N rate increased: N5 had the highest ^15^N excess at the maturity stage in the upper (2.28 ± 0.36%), the middle (1.77 ± 0.28%), and the lower portion (1.68 ± 1.01%). Compared to N1, N fertilization (N2–N5) increased ^15^N excess in the various shoot portions by 50, 38, and 35% at maturity for upper, middle, and lower portions, respectively. At maturity, the ^15^N excess remobilized to the grain under N1–N5 was between 5 and 8%. Our findings revealed that N had a significant impact on yield and N isotope discrimination in spring wheat that these two parameters can interact, and that future research on the relationship between yield and N isotope discrimination in spring wheat should take these factors into account.

## Introduction

Nitrogen (N) is the most important nutrient for plant growth, productivity, and grain quality (Hussain et al., 2020). Optimal use of N fertilizers is essential to improve yield, increase profitability, and minimize environmental consequences from nitrate leaching and nitrous oxide (N_2_O, a greenhouse gas) emissions from denitrification by soil bacteria (Lazcano et al., 2021). The global use of N fertilizer is expected to reach 240 million tons by 2050 (Zhou et al., 2018; Randive et al., 2021). However, plants only use about 40% of the fertilizer N, with the rest escaping into the atmosphere as nitrogenous emissions or getting into groundwater *via* leaching (Nations, 2015).

During crop growth, the plant remobilizes a part of the stored N from the vegetative stem and leaves to reproductive organs such as panicles and grains (Yan et al., 2018; Broberg et al., 2021). N remobilization is genetically controlled (Alpuerto et al., 2021) but could also be influenced by environmental factors (Zheng et al., 2020) and N fertilizer application. The accumulation and relocation of N are considered very significant processes in determining grain yield and grain quality (Gaju et al., 2011; Prey et al., 2019).

The use of ^15^N stable isotope labeling is an excellent method that enables a less biased and more precise estimation of N uptake after anthesis and remobilization of N accumulated during the pre-anthesis period in various organs. N isotope discrimination is controlled by the link between N supply and demand when the N environment is held at a steady-state similar to how carbon isotopes are discriminated during photosynthesis (Dong et al., 2017; Hu and Guy, 2020; Qian et al., 2021). The employment of specialized methodologies, especially for tracking aboveground N dynamics in plants, is required for the precise quantification of N cycling in plant-soil systems (Shan et al., 2012; Meng et al., 2017; Zheng et al., 2018; Xu et al., 2019). Fertilizers can be isotopically labeled with ^15^N to follow its evolution movement in soil-plant systems. Under field conditions, labeling with ^15^N shows promise in analyzing N dynamics from crop residues, which are influenced by the chemical composition and quantity of the crop residue (Berg et al., 1991; Tahir et al., 2018). Handley et al. (2020) defined nitrogen fluxes and ^15^N remobilization during plant development. During the post-anthesis period, the partitioning of the ^15^N assimilated during the pre-anthesis period and remobilized after showed that leaves, stalks, ear, chaff, and sheath serve successively as N sinks and sources. Along with physiological data obtained from model plants (Masclaux-Daubresse and Chardon, 2011; Hawkesford, 2017), N remobilization was shown to be involved in N fluxes from leaf to leaf during the vegetative phase (Wendler, 2018) and from leaves to grains during the reproductive phase. Maintaining yield and profit while minimizing environmental effects requires better fertilizer N management that harmonizes N supply with crop demand (Cui et al., 2010).

Studies have shown that there is a possible interaction between grain isotope discrimination in the aboveground dry matter and grain yield; yet studies on this interaction are few, especially during the crop’s main growth stages (Liang et al., 2013; Chen et al., 2016; Sun et al., 2019). As a result, further information about N isotope discrimination during N uptake and absorption is needed to effectively track the remobilization of N that had been accumulated during the early crop growth stages and the post-anthesis phase under field conditions. However, little has been done in terms of using ^15^N tracing under field conditions to study the effect of N fertilizers on N accumulation and partitioning in spring wheat in the study area. Fewer research efforts have been conducted on how N fertilization affects plant ^15^N (Wang et al., 2016), with previous studies yielding contradicting results (Khalil et al., 2020; Agisho and Hairat, 2021) along with the comparison of N sources being emphasized more frequently (Asif et al., 2020). We hypothesized that the long-term application of inorganic nitrogen fertilizers would impact the relationship between N supply and crop N uptake, and thereby N use efficiency and grain yield. To test this hypothesis, we conducted a study with ^15^N-labeled fertilizer applied to microplots within a long-term trial with five N fertilization rates to study N remobilization, uptake, use efficiency, yield, and post-anthesis losses in spring wheat under rainfed conditions in the semi-arid loess plateau. For determining N remobilization, ^15^N labeling has proven to be a reliable and quantitative method.

## Materials and Methods

### Site Description

**FIGURE 1:**
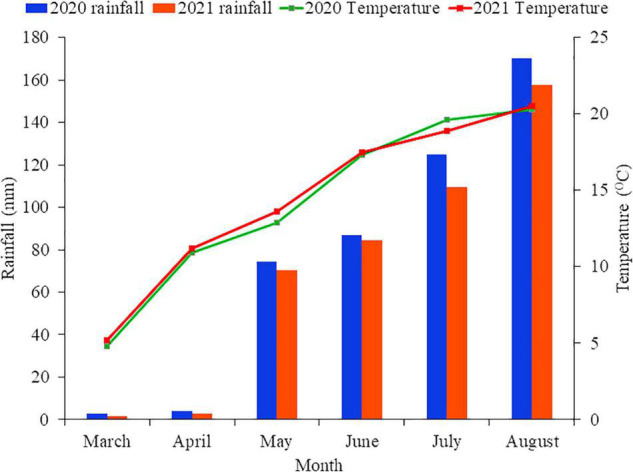
The monthly mean rainfall and temperature during the growing seasons (2020 and 2021) at the experimental site.

This study was conducted in the Gansu Agricultural University’s Rainfed Experimental Station in Dingxi, Gansu Province, China (35°28′N, 104°44′E, elevation 1971 m above sea level) from 2003 to date, but the current study focused on 2020 to 2021 growing seasons (March to July each year). In crop season, the average minimum and maximum air temperatures at the research location (2020 and 2021) were −22 and 38°C, respectively, while the average precipitation was 390.7 mm yr^–1^. With 2,480 h of sunshine for the crop season, the average crop season cumulative air temperature > 10°C was 2240°C, and the average annual radiation was 5,930 MJ m^–2^. The site experienced a rise in the amount of rainfall from May and peaked in August and declined in September (Figure 1). During the growing season, the highest amount of rainfall was recorded in August with 124.9 and 113.5 mm in 2020 and 2021, respectively (Figure 1). Evaporation was three to four times higher than precipitation, with an average of 1,531 mm (coefficient of variation: 24.3%) for the crop season. The soil type at the site is a Huangmian sandy loam (Hocking and Stapper, 2001) and is classified as a Calcaric Cambisol (Hu et al., 2013). Flax (*Linum usitatissimum* L.) had been the previous crop, and the field has a long history of traditional farming with wheat-pea rotation system. The chemical characteristics of the soil (at 0–30 cm depth) were found to be 3.88 kg ha^–1^ of total nitrogen (TN), 24.92 kg ha^–1^ NH_4_-N, 12.72 kg ha^–1^ NO_3_-N, 8.33 pH, 8.3 kg ha^–1^ total phosphorus (P), 4.53 kg ha^–1^ accessible P, and 82.68 kg ha^–1^ total potassium (K).

### Experimental Design and Treatments

The experiment was laid in a randomized complete block design with three replications. The treatments consisted of five N fertilizer (urea) application rates: 0, 52.5, 105, 157.5, and 210 kg ha^–1^, designated as N1, N2, N3, N4, and N5, respectively. In addition, calcium superphosphate to give 105 kg P_2_O_5_ ha^–1^ was applied. The fertilizers were broadcasted across the entire plot area before planting and subsequently mixed into the 0–20 cm soil layer using rotary tillage.

In mid-March, the high-yielding spring wheat (*Triticum aestivum* L.) cultivar “*Dingxi 38*” was planted in rows 20 cm apart at a rate of 187.5 kg seeds ha^–1^ and was harvested in late July to early August. Each test plot measured 30 m^2^ (3 m × 10 m). Within each experimental plot, microplots (0.045 m^2^, 5 plants) were created after seeding. Each plot contained six of these microplots. The microplot was created with a 0.20 m diameter and 0.35 m long polyvinyl chloride (PVC) column that was pushed 0.30 m into the soil at one end of each plot. The microplots, which were spaced 0.5 m apart along the row and between the rows, were used to apply ^15^N-labeled fertilizer and to measure total growth and nitrogen content. During the growing season, weeds were manually removed, and during the fallow periods, after harvesting, Roundup^®^ (glyphosate, 10%) was used to control weeds according to the manufacturer’s instructions. Pests and diseases were monitored and controlled using best practices in the area.

### Plant Sampling and Chemical Analysis

The total growth period was 130 days during 2020 and 128 days during 2021, with six developmental stages recognized (germination, tillering, stem elongation, booting, heading/flowering, and grain-fill/ripening). Ten plants were harvested at 14 days after anthesis (DAA) and separated into ear, leaves, and stem. At maturity, plants were harvested and the biomass was divided into shoots (with chaff) and grains. Dry matter was measured on the different organs of the 10 plants after oven drying for 48 h at 80°C. Before analysis, all samples were ground to a fine powder (<20 mm) in a ball mill. Using the Vapodest 50s (Gerhardt, Königswinter, Germany), the total N concentration in the aboveground biomass was assessed using the Kjeldahl method. Grain yields were recorded for all the treatment after reducing the moisture content to 14%. The N content was determined by multiplying the N concentration with the total biomass (g). The crop’s N uptake was measured at harvest. The amount of N in the straw and grain was multiplied by the dry weight to determine nitrogen uptake.


(1) $$\mathrm{The}\mathrm{ratio}\mathrm{of}N\mathrm{derived}\mathrm{from}\mathrm{fertilizer}\left(\mathrm{Nf}\right)=\left({\mathrm{atom}\%}^{15}N\mathrm{in}\mathrm{organs}\mathrm{treated}\mathrm{by}\mathrm{fertilizer}-0.3663\right)/\left({\mathrm{atom}\%}^{15}N\mathrm{in}\mathrm{fertilizer}-0.3663\right)$$

(2) Namountderivedfromfertilizer=Nf×totalNuptakeamount

(3) Nderivedfromsoil(Ns)=totalNuptakeamount −Nderivedfromfertilizer,

where Nf is the N derived from fertilizer, Ns is N derived from soil, and 0.3663 atom % is the natural abundance of ^15^N (Høgh-Jensen and Schjoerring, 2000).

### ^l5^N Labeling and Analysis

Separate ^15^N enrichment analyses were performed on the ear, leaf, and stem portions at anthesis and additionally on the grain and chaff at maturity. At flowering (June 23, each season), we harvested two of the microplots and 1.68 g m^–2^ N at 10% ^15^N-excess as urea diluted in 1.5 L water were applied on the soil surface of the four remaining microplots. These micro plots were then cut at ground level 14 DAA, and at final harvest (July 29, each season). The N isotope ratio was measured on the different organs using an automatic element analyzer Sercon Control“Callisto CF-IRMS” Version 30.0.11 (Sercon integra 2) coupled to a mass spectrometer (Elemental microanalysis LTD, United Kingdom). The ^15^N enrichment of ^15^N-labeled plant parts fertilized with labeled urea was calculated as an atom percent ^15^N excess adjusted for background abundance (i.e., 0.366%) (Coplen, 2011).

A simple isotopic mass balance mixing equation was used to estimate the average-weight ^15^N values of the aboveground portions of spring wheat plants (Thompson, 1996):


(4) N15=Near15xFear+Nleaves15xFleaves+Nstem15xFstem

(5) $$I=F_{ear}+F_{{}_{leaves}}\text{ + }F_{stem}$$

where ^15^N aboveground, ^15^N ear, ^15^N leaves, and ^15^N stem represent the ^15^N excess (in atom %) for aboveground parts, ear, leaves, and stem of spring wheat, respectively; and F ear, F leaves, and F stem are the total dry weight of spring wheat plant parts ear, leaves, and stem fractions, respectively.


(6) Ni15portion=15NileavesxFileaves+15NistemxFistem

(7) I=Fileaves+Fistem

where ^15^Ni portion is the ^15^N excess (in atom %) for the upper, middle, and lower portion of the shoot of spring wheat plants, respectively, and Fi leaves and Fi stem are the respective leaves and stem fractions in the same portion of the shoot.

The N harvest index (NHI; kg kg^–2^) is the ratio of N in grain to total plant N. The ratio of total dry matter to total nitrogen content was used to calculate total nitrogen utilization efficiency (NUE).

### Statistical Analysis

The data were analyzed using two-way analysis of variance (ANOVA) as implemented in the SPSS software package (Version 17.0) (SPSS Inc., Chicago, IL, United States) to test whether significant differences existed between the treatments and years. *Post hoc* mean separations were done with Duncan Multiple Range Test (DMRT) at a 5% probability level. The results are presented in tables and graphs.

## Results

### Variation in ^15^N Enrichment in Aboveground Components

**FIGURE 2:**
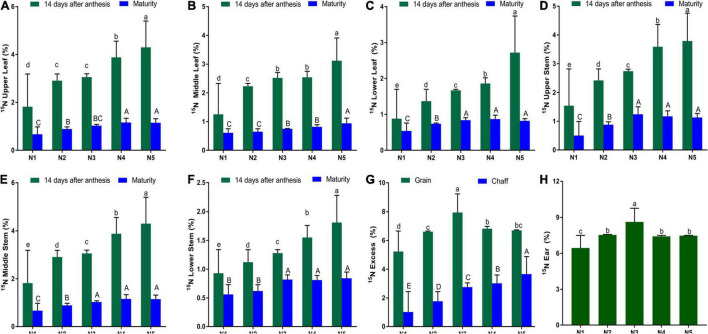
Effect of N fertilization on ^15^N excess (in atom %) of different spring wheat organs with ^15^N labeling. **(A)** Upper leaf. **(B)** Middle leaf. **(C)** Lower leaf. **(D)** Upper stem. **(E)** Middle stem. **(F)** Lower stem. **(G)** Grain and chaff. **(H)** Ear at 14 days after anthesis. Bars with the same color with the same/a common alphabet indicates no significant difference with Duncan Multiple Range Test at 5% probability level and those with different alphabets mean otherwise. Error bars indicate the standard error.

The total ^15^N surplus of leaves and stems was equal among diverse portions of the three shoot portions in both growing seasons. The ^15^N excess of stems and leaves was highest in the upper portion and lowest in the lower portion at maturity (Figure 2). The ^15^N excess of stem and leaf increased with increasing N rate, with N5 recording the highest percentage at the maturity stage for the upper portion (23%), middle portion (24%), and lower portion (22%). However, there were no significant differences between N5, N4, and N3. Compared to the control (N1), N fertilization increased ^15^N excess in the various portions by 50, 38, and 35% at maturity for upper, middle, and lower, respectively (Table 1). Year x N rate interaction had no significant effect on ^15^N excess on the various portions. The percentage ^15^N content of the grain, leaves, stem, and chaff was higher in both growing seasons for all N fertilizer rates compared to the control (Figure 2). In all the wheat plant organs studied, increasing N fertilizer rates resulted in a considerable rise in percentage of ^15^N content. The amount of ^15^N in plant organs increased at an increasing rate at 14 DAA. However, this trend changed at maturity where there was no significant difference between treatments N3 and N5 in terms of ^15^N content of the same organs. The control (N1) obtained the least grain ^15^N content (5%) in wheat at maturity (Figure 2G).

**TABLE 1:** Effect of N fertilizer on isotope values of ^15^N excess (in atom percent) in the upper, middle, and lower portions of shoots in spring wheat.

Year	Treatment	^15^N Upper portion (%)	^15^N Middle portion (%)	^15^N Lower portion (%)
2020	N1	1.06 ± 0.17	1.09 ± 0.03	1.07 ± 0.03
	N2	1.78 ± 0.04	1.38 ± 0.02	1.27 ± 0.04
	N3	2.51 ± 0.18	1.65 ± 0.07	1.69 ± 0.03
	N4	2.52 ± 0.14	1.57 ± 0.02	1.63 ± 0.02
	N5	2.23 ± 0.10	1.67 ± 0.08	1.68 ± 0.04
2021	N1	1.28 ± 0.18	1.10 ± 0.03	1.13 ± 0.01
	N2	1.76 ± 0.07	1.39 ± 0.02	1.46 ± 0.03
	N3	2.04 ± 0.16	1.56 ± 0.05	1.67 ± 0.02
	N4	1.14 ± 0.13	1.66 ± 0.02	1.68 ± 0.03
	N5	2.33 ± 0.16	1.88 ± 0.06	1.64 ± 0.21
MEAN-year	2020	2.02 ± 0.11	1.47 ± 0.05	1.47 ± 0.05
	2021	1.91 ± 0.08	1.52 ± 0.04	1.52 ± 0.03
-N rates	N1	1.17 ± 0.80c	1.10 ± 0.40d	1.10 ± 1.05c
	N2	1.77 ± 0.20b	1.38 ± 0.11c	1.37 ± 0.43b
	N3	2.28 ± 0.31a	1.61 ± 0.11b	1.66 ± 0.25a
	N4	2.33 ± 0.36a	1.62 ± 0.12b	1.66 ± 0.21a
	N5	2.28 ± 0.32a	1.77 ± 0.28a	1.68 ± 1.01a
ANOVA (*F*-value)	Year	0.233^NS^	0.186^NS^	0.107^NS^
	N-rate	<0.001*	<0.001*	<0.001*
	Year × N rate	0.092^NS^	0.024^NS^	0.132^NS^

N rates comprised 0 kg ha^–1^ (N1), 52.5 kg ha^–1^ (N2), 105 kg ha^–1^ (N3), 157.5 kg ha^–1^ (N4), and 210 kg ha^–1^ (N5). Within each level either interaction (year × N-rate), year or N-rate with different alphabets denote significant differences in that level (P < 0.05) with Duncan Multiple Range Test. NS represents non-significant at P > 0.05, while * represents significant at P < 0.05 from ANOVA.

### Effect of N Fertilizer on Crop Biomass and Yield

**FIGURE 3:**
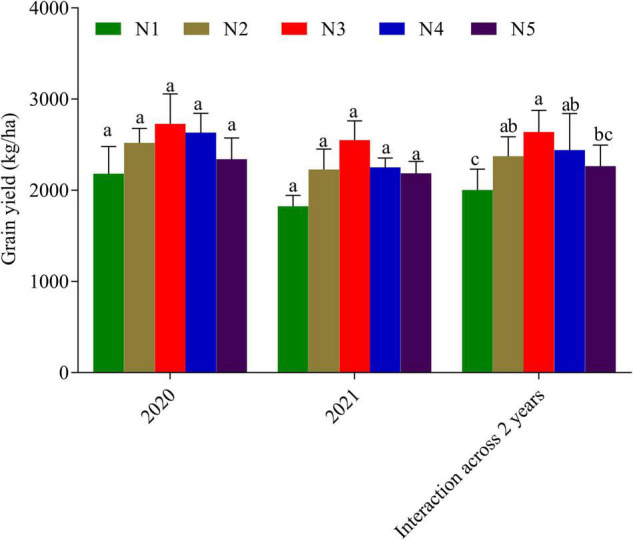
Yield of wheat cultivar fertilized with 0 kg N ha^–1^ (N1), 52.5 kg N ha^–1^ (N2), 105 kg N ha^–1^ (N3), 157 kg N ha^– 1^ (N4), and 210 kg N ha^–1^ (N5). Bars in year/interaction across 2 years with the same/a common alphabet indicates no significant difference with Duncan Multiple Range Test at 5% probability level and those with different alphabets mean otherwise. Error bars indicate the standard error.

Spring wheat grain, straw, and aboveground biomass were all affected by the N rate (Table 2). Straw, grain, and aboveground biomass were all higher in the N3 treatment (5.526 ± 0.62, 4.85 ± 0.93, and 10.371 ± 1.55 t ha^–1^). This was followed by N4 (5.172 ± 0.27, 4.26 ± 0.34, and 9.428 ± 0.61 t ha^–1^, respectively). The least straw, grain, and above ground biomass were as recorded in the control with values of 4.155 ± 0.75, 2.58 ± 1.34, and 6.738 ± 2.08 t ha^–1^, respectively (Table 2). Compared to the control, N fertilization in treatments N3–N5 increased straw, grain, and aboveground biomass by 24.77, 48.04, and 35.01%, respectively. The N rate had a considerable impact on the wheat harvest index (HI). The highest HI was recorded with N3 (0.47 ± 0.03 t ha^–1^) which was not significantly different from N4 and N2 (Table 2). The control (N1) recorded the lowest HI (0.381 ± 0.06 t ha^–1^). Straw, grain, and total aboveground biomass all increased with an increase in the rate of N fertilization. Overall, aboveground dry-matter production was 9.07 ± 0.49 t ha^–1^ in 2020 and 8.58 ± 0.13 t ha^–1^ in 2021 (Table 2). Regardless of the N treatment, the grain yield from fertilized treatments was not significantly different from each other (Figure 3). Although treatment N3 recorded the highest grain yield, the same was not significantly different from the other treatments. When compared to the control, increasing N supply beyond N3 resulted in a negligible yield gain (Figure 3). This suggests that increasing N beyond N3 may not be economical.

**TABLE 2:** Effects of N rate on grain, straw, and aboveground biomass; harvest index (HI), nitrogen harvest index (NHI), and use efficiency (NUE) of spring wheat cultivar.

Year	Treatment	Straw dry matter yield (t ha^–1^)	Grain dry matter yield (t ha^–1^)	Aboveground dry matter (t ha^–1^)	Harvest index	NHI (kg kg^–1^)	NUE (%)
2020	N1	4.22 ± 0.02	2.84 ± 0.08	7.06 ± 0.08	0.40 ± 0.01	0.87 ± 0.03	26.51
	N2	4.83 ± 0.03	4.52 ± 0.23	9.35 ± 0.22	0.48 ± 0.04	0.81 ± 0.01	20.40
	N3	5.61 ± 0.05	4.93 ± 0.10	10.54 ± 0.08	0.47 ± 0.02	0.77 ± 0.01	16.68
	N4	5.21 ± 0.04	4.34 ± 0.09	9.56 ± 0.12	0.45 ± 0.01	0.74 ± 0.02	17.73
	N5	4.96 ± 0.03	3.86 ± 0.11	8.82 ± 0.11	0.44 ± 0.01	0.81 ± 0.01	18.68
2021	N1	4.09 ± 0.01	2.32 ± 0.07	6.42 ± 0.07	0.36 ± 0.01	0.81 ± 0.01	26.57
	N2	4.72 ± 0.02	3.70 ± 0.22	8.42 ± 0.23	0.44 ± 0.01	0.82 ± 0.01	20.13
	N3	5.44 ± 0.03	4.76 ± 0.09	10.20 ± 0.09	0.47 ± 0.04	0.76 ± 0.02	15.79
	N4	5.13 ± 0.04	4.17 ± 0.11	9.30 ± 0.12	0.45 ± 0.01	0.77 ± 0.02	17.56
	N5	4.82 ± 0.02	3.72 ± 0.12	8.54 ± 0.10	0.43 ± 0.01	0.82 ± 0.01	19.94
Year	2020	4.97 ± 0.13	4.10 ± 0.36	9.07 ± 0.49	0.45 ± 0.02	0.80 ± 0.01	19.99
	2021	4.84 ± 0.04	3.74 ± 0.12	8.58 ± 0.13	0.43 ± 0.01	0.79 ± 0.02	20.01
N-rate	N1	4.16 ± 0.75d	2.58 ± 1.34d	6.74 ± 2.08d	0.38 ± 0.06c	0.84 ± 0.04a	26.54
	N2	4.77 ± 0.13c	4.11 ± 0.19bc	8.89 ± 0.06c	0.46 ± 0.02ab	0.82 ± 0.02a	20.27
	N3	5.53 ± 0.62a	4.85 ± 0.93a	10.37 ± 1.55a	0.47 ± 0.03a	0.76 ± 0.03b	16.23
	N4	5.17 ± 0.27b	4.26 ± 0.34b	9.43 ± 0.61b	0.45 ± 0.01ab	0.76 ± 0.04b	17.65
	N5	4.89 ± 0.01c	3.79 ± 0.12c	8.68 ± 0.14c	0.44 ± 0.01b	0.82 ± 0.02a	19.31
ANOVA (*P*-value)	Year	0.004*	0.008*	0.002*	0.039*	0.676^NS^	0.001*
	N-rate	<0.001*	<0.001*	<0.001*	<0.001*	<0.001*	<0.001*
	Year × N rate	0.966^NS^	0.356^NS^	0.450^NS^	0.303^NS^	0.074^NS^	0.350^NS^

N rates comprised 0 kg ha^–1^ (N1), 52.5 kg ha^–1^ (N2), 105 kg ha^–1^ (N3), 157.5 kg ha^–1^ (N4), and 210 kg ha^–1^ (N5). Within each level either interaction (year × N-rate), year or N-rate with different alphabets denote significant differences in that level (P < 0.05) with Duncan Multiple Range Test. NS represents non-significant at P > 0.05, while * represents significant at P < 0.05 from ANOVA.

### Effect of N Rates on N Concentration and Uptake

The grain N concentration was higher in the high N treatments at N3, N4, and N5 (19.66 ± 1.57, 18.65 ± 0.55, and 18.00 ± 0.10 g kg^–1^, respectively) than in N1 (16.25 g kg^–1^) (Table 3). A similar trend was observed for the concentration of N in straw 5.30 ± 1.55, 4.91 ± 1.17, and 3.12 ± 0.62 g kg^–1^, respectively, for N3, N4, and N5. With increasing N rate, wheat N concentration and uptake increased (Table 3). The N rate had a significant effect on wheat N uptake for both grain and straw. The N3 treatment had the highest N uptake in straw (29.27 ± 10.38 kg ha^–1^) and in grain (95.27 ± 23.49 kg ha^–1^) compared to the other treatments. Wheat uptake of total nitrogen ranged between 7.88 and 29.27 kg ha^–1^ for straw and 41.85 and 95.27 kg ha^–1^ for grain (Table 3). Relative to N1, N fertilization increased the N content in straw by 73.1% and in grain by 56.1%.

**TABLE 3:** Effects of N fertilizer rate on N accumulation and uptake in grain and straw of spring wheat cultivar.

Year	Treatment	Straw N concentration (g kg^–1^)	Grain N concentration (g kg^–1^)	Straw N Uptake (kg ha^–1^)	Grain N Uptake (kg ha^–1^)
2020	N1	1.66 ± 0.40	16.50 ± 0.02	7.01 ± 1.96	46.79 ± 0.74
	N2	3.97 ± 0.32	18.17 ± 0.01	19.13 ± 1.43	82.13 ± 4.22
	N3	5.23 ± 0.23	19.93 ± 0.03	29.36 ± 1.01	98.19 ± 1.29
	N4	5.36 ± 0.28	18.76 ± 0.13	27.96 ± 1.40	81.64 ± 2.14
	N5	3.33 ± 0.28	18.33 ± 0.09	16.43 ± 0.13	71.20 ± 1.53
2021	N1	2.14 ± 0.42	16.01 ± 0.03	8.74 ± 1.99	36.91 ± 0.81
	N2	3.00 ± 0.30	17.66 ± 0.01	14.09 ± 1.40	65.28 ± 3.89
	N3	5.36 ± 0.22	19.39 ± 0.02	29.19 ± 1.03	92.35 ± 1.23
	N4	4.47 ± 0.26	18.54 ± 0.08	22.97 ± 1.41	77.50 ± 2.06
	N5	2.91 ± 0.29	17.67 ± 0.11	13.99 ± 0.42	65.85 ± 1.62
MEAN-year	2020	3.91 ± 0.34a	18.34 ± 0.48a	19.98 ± 2.18a	75.99 ± 2.09a
	2021	3.58 ± 0.19a	17.85 ± 0.32a	17.80 ± 0.92a	67.58 ± 3.85b
-N rate	N1	1.900 ± 1.84c	16.25 ± 1.84c	7.88 ± 11.01d	41.85 ± 29.93d
	N2	3.484 ± 0.26b	17.91 ± 0.18b	16.61 ± 2.28c	73.70 ± 1.92bc
	N3	5.298 ± 1.55a	19.66 ± 1.57a	29.27 ± 10.38a	95.27 ± 23.49a
	N4	4.914 ± 1.17a	18.65 ± 0.55ab	25.46 ± 6.58b	79.57 ± 7.79b
	N5	3.120 ± 0.62b	18.00 ± 0.10b	15.21 ± 3.68c	68.53 ± 3.26c
ANOVA (*P*-value)	Year	0.086^NS^	0.152^NS^	0.030^NS^	0.006
	N-rate	<0.001*	<0.001*	<0.001*	<0.001*
	Year × N rate	0.096^NS^	0.995^NS^	0.126^NS^	0.574^NS^

N rates comprised 0 kg ha**^–^**^1^ (N1), 52.5 kg ha**^–^**^1^ (N2), 105 kg ha**^–^**^1^ (N3), 157.5 kg ha**^–^**^1^ (N4), and 210 kg ha**^–^**^1^ (N5). Within each level either interaction (year × N-rate), year or N-rate with different alphabets denote significant differences in that level (P < 0.05) with Duncan Multiple Range Test. NS represents non-significant at P > 0.05, while * represents significant at P < 0.05 from ANOVA.

### Delivery of ^15^N and Soil N in Wheat Cultivar

In both seasons, Nf in wheat grain and straw increased while Ns declined as the N rate was increased. The total grain of wheat N derived from ^15^N fertilizer (44.19–73.84%) increased with the increased N application rate (*P* < 0.001) (Table 4). Total straw N derived from fertilizer ranged between 7.14 and 15.80%. Wheat grain N derived from soil N ranged between 21.43 and 28.57%, while that of straw N derived from soil N ranged between 6.78 and 13.47%. There was a significant difference between years by N rate interaction for all parameters (Nf Grain, Ns Grain, and Ns straw) except Ns straw (Table 4).

**TABLE 4:** Effect of N fertilizer rates on N derived from fertilizer and soil at maturity in 2020–2021.

Year	Treatment	Nf Grain (%)	Nf Straw (%)	Total (%)	Ns Grain (%)	Ns Straw (%)	Total (%)
2020	N1	-	-	-	-	-	-
	N2	49.07 ± 1.91e	8.07 ± 0.07d	55.77	33.06 ± 2.87a	11.06 ± 1.01c	44.12
	N3	75.36 ± 0.51a	16.72 ± 0.08a	92.08	22.83 ± 0.22d	14.30 ± 1.41a	37.13
	N4	53.98 ± 0.70c	15.56 ± 0.68b	69.54	27.66 ± 0.88b	12.40 ± 0.23b	40.06
	N5	45.52 ± 0.70f	8.90 ± 0.53d	54.42	25.68 ± 1.78c	7.53 ± 0.17d	33.21
2021	N1	-	-	-	-	-	-
	N2	41.19 ± 1.92h	6.22 ± 0.08e	47.41	24.09 ± 2.85d	7.87 ± 0.98d	31.96
	N3	72.32 ± 0.50b	14.89 ± 0.06b	87.21	20.03 ± 0.23e	12.64 ± 1.39b	32.67
	N4	51.32 ± 0.71d	12.20 ± 0.65c	63.52	26.18 ± 0.89c	10.77 ± 0.25c	36.95
	N5	42.85 ± 0.71g	7.97 ± 0.52d	50.82	26.00 ± 1.76c	6.02 ± 0.15e	32.02
MEAN-year	2020	55.98 ± 4.06a	12.31 ± 1.99a	68.29	27.31 ± 3.23a	10.91 ± 1.17a	38.22
	2021	51.92 ± 0.32b	10.32 ± 0.26b	62.24	24.07 ± 0.32b	9.74 ± 0.26b	33.81
-N rate	N1	-	-	-	-	-	-
	N2	45.13 ± 8.82c	7.14 ± 4.17d	52.27	28.57 ± 2.88a	9.47 ± 0.86c	38.04
	N3	73.84 ± 19.89a	15.80 ± 4.49a	89.64	21.43 ± 4.26d	13.47 ± 3.15a	34.90
	N4	52.65 ± 1.30b	13.88 ± 2.56b	66.53	26.92 ± 1.23b	11.59 ± 1.26b	38.51
	N5	44.19 ± 9.76c	8.43 ± 2.88c	52.62	25.84 ± 0.15c	6.78 ± 5.55d	32.62
ANOVA (*P*-value)	Year	<0.001*	<0.001*	<0.001*	<.001*	<0.001*	<0.001*
	N-rate	<0.001*	<0.001*	<0.001*	<.001*	<0.001*	<0.001*
	Year × N rate	<0.001*	0.036^NS^	<0.001*	<.001*	<0.001*	<0.001*

N rates comprised 0 kg ha**^–^**^1^ (N1), 52.5 kg ha**^–^**^1^ (N2), 105 kg ha**^–^**^1^ (N3), 157.5 kg ha**^–^**^1^ (N4), and 210 kg ha**^–^**^1^ (N5). Within each level either interaction (year × N-rate), year or N-rate with different alphabets denote significant differences in that level (P < 0.05) with Duncan Multiple Range Test. NS represents non-significant at P > 0.05, while * represents significant at P < 0.05 from ANOVA.

### Remobilization of Labeled Nitrogen Fertilizer (Urea)

The remobilization of labeled N fertilizer differed significantly among the treatments and was affected by N fertilization. The highest remobilization of ^15^N excess to the grain was recorded by N3 (7.95%), which was significantly different from the other treatments. At maturity, the percentage of ^15^N excess remobilized to the grain was 5.23, 6.61, 7.95, 6.82, and 6.69% for the treatments N1, N2, N3, N4, and N5, respectively (Table 4). Remobilization of ^15^N to the ears (grain and chaff) accounted for 48.33% of post-anthesis stored N in N3. Compared to the control N, fertilization increased ^15^N by 34.2% (Table 4). ^15^N fertilizer remobilization did not differ from year to year. This implies that the results were relatively consistent in each year. A large amount of early-accumulated ^15^N was unaccounted for, and it was presumed that it had been lost. Loss of accumulated ^15^N at 14 DAA increased with increasing N fertilization (Table 4). The highest loss occurred in N5 and N4 with 10.03 ± 3.01 and 7.98 ± 0.97, respectively, representing 38.4 and 34.1% of the total accumulated ^15^N at 14 DAA.

## Discussion

The N distribution in different plant parts, grain yield as well as N uptake of spring wheat fertilized with different amounts of N were studied in this work. Application of N fertilizer increased the ^15^N content of the various aboveground component of spring wheat cultivar under field growing conditions. During the grain filling stage and maturity, there were considerable differences in ^15^N excess among wheat aboveground components; the most significant difference, between N1 and N5, was 57.67% for upper leaf and 34.29% for grain (Figure 2). These findings imply that N fertilization was more important for grain filling in wheat plants during the grain filling stage. Our findings are consistent with that of Ba et al. (2020) who reported that the redistribution of labeled N from source organs (flag leaves and stem) to sink organs (ears) was more pronounced in high N wheat plants. These differences were caused by the fact that N remobilization begins earlier in plants under low N fertilization conditions than it does under high N fertilization conditions (Aranjuelo et al., 2013). In spring wheat, the ^15^N enrichment of shoots and ears varied according to developmental stages.

In our present study, when compared to other aboveground components, spring wheat ears accumulate more ^15^N during the grain filling stage (14 DAA) (Figure 2G). The concentration of ^15^N in the ear did not decrease much with time (14 DAA-maturity) as it did in stem and leaves, except under N1 and N2 (Figure 2). Similar effects have been observed in sorghum (He et al., 2022). The ears serve as a strong N sink, impacting N accumulation in the ears throughout grain filling, whereas the shoots provide nutrients to the ears (Aranjuelo et al., 2013; Zhou et al., 2016; Sun et al., 2018).

In our study, the grain yield of the spring wheat cultivar was 11.52–24.13% greater with the N treatment (N2–N5) than with the control (N1). N treatments, on the other hand, had no significant effect on grain yield (Figure 3). When the N rate was increased from 105 to 210 kg ha^–1^, wheat grain yield did not differ significantly (Figure 3). This could be explained by the fact that the study site has been fertilized continuously for a long time. In some studies, N fertilizer application has proven to boost crop yield (Abad et al., 2004), while excessive and long-term N fertilizer use has resulted in yield reduction (Garrido-Lestache et al., 2005; Wang et al., 2011; Ierna et al., 2015; Agegnehu et al., 2016).

Since water availability is the major factor limiting grain yield, the overall biomass yield in 2020 was higher than in 2021, a result that was linked to less rainfall throughout the growing period in 2021 (Figure 1; Selles and Zentner, 2001). The current study found that increasing the N rate enhanced grain, straw, and aboveground biomass of spring wheat (*P* < 0.001), as found by other researchers (Wang et al., 2010; Zhao et al., 2014). The spring wheat cultivar had a lower HI in the control. However, there were no significant differences between the N treatments. This may be attributed to the fact that increased N-rate-enhanced wheat development in the early stages than the control treatment (Mahler et al., 1994). Our results appeared to agree with those of Mahjourimajd et al. (2016), who observed that a different N rate supply did not influence HI in an Australian wheat mapping population.

Increasing N application rates improved both grain (17.91–19.66 g kg^–1^) and straw (3.12–5.29 g kg^–1^) N concentration of spring wheat in our present field experiment, with the best effect observed in N3 in both growing seasons (Table 3). Chen et al. (2016) reported a similar pattern that while increasing the N application rate from 60 to 240 kg ha^–1^, the N concentration increased from 2 to 4 and 15 to 22 g kg^–1^ for straw and grain, respectively. Post-anthesis nitrogen uptake is an important parameter for identifying higher-yield wheat varieties (Monaghan et al., 2001), which allowed researchers to distinguish the contrasting behavior between wheat varieties under varying N supply. Results from our study showed that the N fertilizer rate had a significant effect on N uptake by grain and straw. This may be attributed to enhanced biomass yield and N concentration of the N treatments when compared to the control (Table 3). The lower NUE values recorded at maturity (Table 2) in our present study is an indication that excessive and continuous application of N fertilizer does not only promote vegetative growth but also reduces plants’ ability to utilize nutrients under field conditions. According to the “law of diminishing returns,” a high N application rate indicates a low NUE under normal conditions. Crop N uptake, in addition to fertilizer N rate, is another direct factor that affects NUE (Zhang et al., 2021).

Our findings revealed that, when the rate of applied N increased, the amount of early stored ^15^N remobilized to both ears and grain increased as also observed by previous research efforts (Palta and Fillery, 1995; Zhou et al., 2018). They suggested that the rate of applied N increased the demand for early stored N, probably because the increased early availability of N increased the size of the sink. The rate at which N was applied affected the loss of pre-anthesis stored nitrogen during post-anthesis. Between 31 and 38% of the ^15^N in the crop at the grain filling stage (14 DAA) had been lost by maturity at N1–N5, and the entire excess N that remained in the crop was remobilized to the grain, resulting in an absolute increase in pre-anthesis stored N transferred to the grain. Another important finding of this study was that increasing the rate of applied N increased post-anthesis N losses in spring wheat (Table 4). However, N3 compared to the other treatments (N2, N4, and N5) recorded the least N loss, indicating that at this rate the plant can remobilize a greater portion of the applied N (Table 5). Shivay et al. (2020) observed that N application rate and year have an effect on N losses in spring wheat and suggested ammonia volatilization from the aerial parts of the plants as the major source. Higher N losses from the plant are attributable to higher N expenditure during the remobilization process (O’Deen, 1989; Reining et al., 1995; Sra et al., 2004). Losses from the crop need to be explored further, and attention must be drawn to the crop as more than simply a sink for N. The N recovery for wheat in the Middle and Lower Yangtze River Region was reported to be between 33.0 and 49.0% by some researchers (Zhao et al., 2009; Shi et al., 2012). Xiao-Tang et al. (2007) found that the N recovery in wheat was 54% at 120 kg N ha^–1^ and 32% at 360 kg N ha^–1^, and it is affected by wheat cultivars, soil fertility, and climate conditions (Wang et al., 2011), a result that is similar to our present study.

**TABLE 5:** Post-anthesis effect of N fertilization on ^15^N accumulated by the grain filling stage (14 DAA), losses and remobilization to the ears and grain between 14 DAA and maturity for spring wheat cultivar.

Year	Treatment	15N 14 DAA (mg m^2^)	Remobilized Grain	Remobilized Chaff	Retained in Straw	Post-anthesis losses
2020	N1	14.18 ± 0.09	5.20 ± 0.10	1.04 ± 0.06	3.22 ± 0.15	4.72 ± 0.17
	N2	19.87 ± 0.05	6.78 ± 0.10	1.80 ± 0.04	4.43 ± 0.10	6.87 ± 0.05
	N3	22.29 ± 0.10	7.98 ± 0.03	2.77 ± 0.03	5.85 ± 0.28	5.69 ± 0.35
	N4	23.99 ± 0.31	6.90 ± 0.01	3.06 ± 0.02	5.72 ± 0.12	8.30 ± 0.17
	N5	26.27 ± 0.08	6.76 ± 0.09	3.69 ± 0.01	5.58 ± 0.14	10.24 ± 0.06
2021	N1	13.85 ± 0.10	5.26 ± 0.07	1.00 ± 0.05	3.51 ± 0.13	4.08 ± 0.18
	N2	19.48 ± 0.06	6.44 ± 0.05	1.75 ± 0.04	4.61 ± 0.09	6.67 ± 0.06
	N3	21.98 ± 0.09	7.91 ± 0.10	2.72 ± 0.03	5.28 ± 0.25	6.08 ± 0.34
	N4	22.86 ± 0.28	6.74 ± 0.06	2.97 ± 0.02	5.48 ± 0.11	7.66 ± 0.15
	N5	25.93 ± 0,07	6.63 ± 0.03	3.63 ± 0.01	5.85 ± 0.15	9.81 ± 0.05
MEAN-year	2020	21.32 ± 0.50	6.72 ± 0.13	2.47 ± 0.06	4.96 ± 0.01	7.16 ± 0.30
	2021	20.82 ± 0.16	6.60 ± 0.05	2.41 ± 0.03	4.95 ± 0.09	6.86 ± 0.22
-N rate	N1	14.01 ± 7.06e	5.23 ± 1.43d	1.02 ± 1.42e	3.37 ± 1.59c	4.40 ± 2.62e
	N2	19.67 ± 1.40d	6.61 ± 0.05c	1.77 ± 0.67d	4.52 ± 0.43b	6.77 ± 0.24d
	N3	22.14 ± 1.07c	7.95 ± 1.28a	2.75 ± 0.30c	5.56 ± 0.61a	5.89 ± 1.13c
	N4	23.42 ± 2.35b	6.82 ± 0.16b	3.02 ± 0.58b	5.60 ± 0.65a	7.98 ± 0.97b
	N5	26.10 ± 5.03a	6.69 ± 0.04bc	3.66 ± 1.22a	5.72 ± 0.76a	10.03 ± 3.01a
ANOVA (*P*-value)	Year	0.005*	0.031^NS^	0.070^NS^	0.888^NS^	0.191^NS^
	N-rate	<0.001*	<0.001*	<0.001*	<0.001*	<0.001*
	Year × N rate	0.438^NS^	0.267^NS^	0.992^NS^	0.023^NS^	0.579^NS^

N rates comprised 0 kg ha**^–^**^1^ (N1), 52.5 kg ha**^–^**^1^ (N2), 105 kg ha**^–^**^1^ (N3), 157.5 kg ha**^–^**^1^ (N4), and 210 kg ha**^–^**^1^ (N5). Within each level either interaction (year × N-rate), year or N-rate with different alphabets denote significant differences in that level (P < 0.05) with Duncan Multiple Range Test. NS represents non-significant at P > 0.05, while * represents significant at P < 0.05 from ANOVA.

## Conclusion

In conclusion, this study found that NUE strength in the study area is relatively low and that it has to be improved in spring wheat cultivation by using appropriate fertilization strategies. Our results showed that soil with low fertility (N1 and N2) treatments had the lowest grain yield and straw and grain dry matter which is an indication that N fertilization affects yield and its attributes. Long-term and excessive N application (210 kg ha^–1^) reduces grain yield and increases N loss in a wheat crop under the conditions of this study. The upper portions of leaves and stem were more strongly enriched with ^15^N than the lower (aged) ones at both 14 DAA and maturity which is an indication that the upper portion of the leaves, in particular, maybe a great sink and storage for N in the plant during development, as well as a major sink for N freshly taken up post-anthesis. The importance of the ear as a sink for nitrogen is shown by the fact that a greater percentage of total plant nitrogen at 14 DAA was found in the ear, which accounted for a chunk of total dry matter. The results from this study offer useful insights for N application in spring-cultivated wheat in an attempt to reduce the quantity of N fertilizer inputs, thereby costs, and in producing wheat in an environmentally sustainable manner.

## Data Availability Statement

The original contributions presented in the study are included in the article/supplementary material, further inquiries can be directed to the corresponding author.

## Author Contributions

LL: funding acquisition, conceptualization, and supervision. JW: resources and project administration. ZE: investigation and writing—original draft. JX: methodology. ZE, JW, and MZ: data collection. ZE, SB, and BK: formal analysis. LL, BK, and JP: writing—review and editing. All authors read and approved the final manuscript.

## Conflict of Interest

The authors declare that the research was conducted in the absence of any commercial or financial relationships that could be construed as a potential conflict of interest.

## Publisher’s Note

All claims expressed in this article are solely those of the authors and do not necessarily represent those of their affiliated organizations, or those of the publisher, the editors and the reviewers. Any product that may be evaluated in this article, or claim that may be made by its manufacturer, is not guaranteed or endorsed by the publisher.
